# Effects of grain intervention on hypothalamic function and the metabolome of blood and milk in dairy cows

**DOI:** 10.1186/s40104-024-01034-3

**Published:** 2024-06-01

**Authors:** Limei Lin, Kaizhen Guo, Huiting Ma, Jiyou Zhang, Zheng Lai, Weiyun Zhu, Shengyong Mao

**Affiliations:** 1 Laboratory of Gastrointestinal Microbiology, Jiangsu Key Laboratory of Gastrointestinal Nutrition and Animal Health, National Center for International Research On Animal Gut Nutrition, College of Animal Science and Technology, Nanjing Agricultural University, Nanjing, 210095 China; 2 Ruminant Nutrition and Feed Engineering Technology Research Center, College of Animal Science and Technology, Nanjing Agricultural University, Nanjing, 210095 China

**Keywords:** Blood, Grain-based diet, Hypothalamus, Metabolomics, Milk, Prostaglandin E_2_, Transcriptomics

## Abstract

**Background:**

The hypothalamus plays a crucial role in the health and productivity of dairy cows, yet studies on its functionality and its impact on peripheral circulation in these animals are relatively scarce, particularly regarding dietary interventions. Therefore, our study undertook a comprehensive analysis, incorporating both metabolomics and transcriptomics, to explore the effects of a grain-based diet on the functionality of the hypothalamus, as well as on blood and milk in dairy cows.

**Results:**

The hypothalamic metabolome analysis revealed a significant reduction in prostaglandin E_2_ (PGE_2_) level as a prominent response to the grain-based diet introduction. Furthermore, the hypothalamic transcriptome profiling showed a notable upregulation in amino acid metabolism due to the grain-based diet. Conversely, the grain-based diet led to the downregulation of genes involved in the metabolic pathway from lecithin to PGE_2_, including phospholipase A2 (*PLA2G4E*, *PLA2G2A*, and *PLA2G12B*), cyclooxygenase-2 (*COX2*), and prostaglandin E synthase (*PTGES*). Additionally, the plasma metabolome analysis indicated a substantial decrease in the level of PGE_2_, along with a decline in adrenal steroid hormones (tetrahydrocortisol and pregnenolone) following the grain-based diet introduction. Analysis of the milk metabolome showed that the grain-based diet significantly increased uric acid level while notably decreasing PGE_2_ level. Importantly, PGE_2_ was identified as a critical metabolic marker in the hypothalamus, blood, and milk in response to grain intervention. Correlation analysis demonstrated a significant correlation among metabolic alterations in the hypothalamus, blood, and milk following the grain-based diet.

**Conclusions:**

Our findings suggest a potential link between hypothalamic changes and alterations in peripheral circulation resulting from the introduction of a grain-based diet.

**Supplementary Information:**

The online version contains supplementary material available at 10.1186/s40104-024-01034-3.

## Background

Dairy cows have the ability to convert low-quality lignocellulose into high-nutrient milk, relying not only on the efficient degradation of complex polysaccharides by gastrointestinal microbiota but also on the maintenance and regulation of host homeostasis [1, 2]. Critical to this process is the bidirectional communication between the brain and peripheral organs, essential for sustaining homeostasis. The hypothalamus, a key brain region, is instrumental in maintaining internal balance, regulating the endocrine system, and adapting to external environmental shifts [3, 4]. Previous studies in dairy cows have shown that the hypothalamus can influence feeding behavior, energy metabolism, and peripheral inflammation through mechanisms such as the vagus nerve and humoral pathways [5, 6]. Therefore, exploring hypothalamic functionality is of significant importance for improving milk yield and maintaining host health in dairy cows.

To enhance milk production and meet the demand for dairy products, a gradual transition from the traditional forage-based diets to grain-based diets for dairy cows on commercial farms is a common agricultural practice [7–9]. However, the potential impact of long-term grain-based diet feeding on dairy cows cannot be underestimated, particularly with regard to its effects on peripheral tissues [10–12], leading to a greater susceptibility to various metabolic disorders in dairy cows [13]. Despite the acknowledged significance of hypothalamic functionality in homeostatic regulation, research regarding the impact of grain-based diets on the hypothalamus is still lacking. Studies on the effects of factors like heat stress [14], restricted feeding [15], and artificial stress [16] on hypothalamic tissues have shown that changes in hypothalamic function can lead to alterations in the hypothalamic–pituitary–adrenal (HPA) axis, resulting in hormonal fluctuations in the peripheral circulation and shifts in energy metabolism. Therefore, exploring the effects of grain-based diets on hypothalamic tissues is of significant importance for the subsequent regulation of peripheral circulation.

To address this gap, we employed untargeted liquid chromatography–mass spectrometry (LC–MS) and transcriptome sequencing on hypothalamus of Holstein cows fed with forage-based versus grain-based diets to explore the effects of grain intervention on hypothalamic functions. Additionally, LC–MS analysis on plasma and milk was conducted to explore metabolic changes in the blood and milk, thereby elucidating the relationship between the hypothalamus and the peripheral circulation following the introduction of a grain-based diet in dairy cows.

## Methods

### Animals and experimental design

Our study selected 12 healthy, multiparous (second calving), late-lactation Holstein cows, each approximately 3 years old and averaging a body weight of 651 ± 54 kg. These cows were individually housed in tie stalls for a one-month study duration. All cows were in good condition, with body condition scores of 3.0 to 3.5 (5-point scale) [17]. Prior to the experimental period, all dairy cows were initially provided with a forage-based diet with a forage-to-concentrate ratio of 6:4 on a dry matter basis for one week (as detailed in Additional file 1: Table S1). After this adaptation period, the cows were randomly divided into two groups: the F group (comprising 6 cows) continued to be fed the forage-based diet, while the G group (also 6 cows) transitioned to a grain-based diet with a forage-to-concentrate ratio of 4:6 on a dry matter basis (as specified in Additional file 1: Table S1). During the 2 d preceding the experimental period, a gradual dietary transition was executed, with the concentrate portion of the feed being increased by 10% daily for cows in the G group. The days in milk (F, 232.8 ± 17.0; G, 233.7 ± 16.4) and milk yield (F, 17.0 ± 4.2 kg/d; G, 17.8 ± 3.9 kg/d) were consistent across both groups of cows (*P* > 0.05; Additional file 2: Fig. S1A and S1B). The experimental period spanned 21 d, during which the animals were provided with ad libitum access to food (5%–10% refusals), with feedings occurring twice daily at 07:00 and 19:00.

### Sampling scheme

Throughout the experiment, feed supplied and feed residues for each cow were measured and documented on the final 2 days of each week. Additionally, samples of fresh feed and leftovers for each cow were taken daily and stored at –20 °C for later analysis. The dry matter content in the feed was evaluated by drying in an oven at 55 °C for 72 h. Two days before slaughter, the cows were milked twice daily at 06:30 and 18:30 h for untargeted LC–MS analysis. On the final day of the experiment, all the animals were humanely euthanized, and their internal organs were promptly dissected. Tail vein blood samples were collected during the 2 d leading up to slaughter after the morning feeding. The blood samples were immediately centrifuged at 3,000 × *g* for 10 min to obtain plasma samples. These obtained plasma samples were then utilized for untargeted LC–MS analysis. Hypothalamic tissues were rapidly dissected using a sterile blade on ice and preserved in liquid nitrogen for subsequent LC–MS analysis and RNA extraction.

### LC–MS analysis

For hypothalamic tissue samples, precisely weighed freeze-dried samples (50 mg) were transferred to a centrifuge tube. Then, 800 μL of 80% methanol and 5 μL of DL-o-Chlorophenylalanine (2.8 mg/mL) were added, followed by 30 s of vortex mixing at 65 kHz for 90 s. The mixture was sonicated for 30 min at 4 °C, and subsequently left to stand at –20 °C for 1 h. After centrifugation at 4 °C and 12,000 r/min for 15 min, 200 μL of the supernatant was collected in a vial for LC–MS analysis.

For plasma and milk samples, 100 μL of each sample was taken in a centrifuge tube. Subsequently, 300 μL of methanol and 10 μL of the internal standard DL-o-Chlorophenylalanine (2.8 mg/mL) were added, followed by 30 s of vortex mixing. The mixture was left to stand at –20 °C for 1 h. Afterward, it was centrifuged at 12,000 r/min and 4 °C for 15 min, and 200 μL of the supernatant was collected in a vial for LC–MS analysis.

After preprocessing of the hypothalamus tissue, plasma, and milk samples, 10 μL of each sample was taken for injection and subjected to LC–MS analysis on a Hyper gold C18 column (100 mm × 2.1 mm, 1.9 μm). The specific chromatographic separation conditions included column temperature of 40 °C, flow rate of 0.3 mL/min, and mobile phase A (water + 5% acetonitrile + 0.1% formic acid) and mobile phase B (acetonitrile + 0.1% formic acid), automatic injector temperature of 4 °C. Both electrospray ionization (ESI) in positive and negative modes was utilized for MS data acquisition. The following parameters were employed: Heater Temperature set to 300 °C, Sheath Gas Flow Rate at 45 arb, Auxiliary Gas Flow Rate at 15 arb, Sweep Gas Flow Rate at 1 arb, a spray voltage of 3.0 kV for positive mode and 3.2 kV for negative mode, Capillary Temperature maintained at 350 °C, with the S-Lens RF Level set at 30% for positive mode and 60% for negative mode. We then performed data extraction and pre-processing of LC–MS detection data using the Compound Discoverer software (Thermo Fisher). Full-spectrum identification was conducted based on mass values in the Excel data sheet and matched against standard metabolome databases including the Human Metabolome Database (https://hmdb.ca) [18], MassBank (https://massbank.jp) [19], and Metlin (https://metlin.scripps.edu) [20]. The sparse partial least squares discriminant analysis (sPLS-DA) was applied to the complete set of metabolites. Visual representation of the data included 95% confidence interval ellipses for each group, which were based on the standard deviation. Furthermore, metabolic pathway analysis was conducted using MetaboAnalyst [21] (v.5.0).

### RNA extraction, library preparation, and Illumina sequencing

The hypothalamic tissues were subjected to RNA extraction using TRIzol reagent (Invitrogen, Carlsbad, CA, USA) following the manufacturer’s guidelines, and any genomic DNA was removed with DNase I (TaKaRa Bio, Otsu, Japan). The RNA quality was assessed using a 2100 Bioanalyzer (Agilent Technologies, Palo Alto, CA, USA), and RNA concentration was determined with an ND-2000 spectrophotometer (NanoDrop Technologies, Wilmington, DE, USA). High-quality RNA (2 μg/sample) was processed according to the TruSeq^TM^ RNA sample preparation kit (Illumina, San Diego, CA, USA), which included mRNA isolation, fragmentation, cDNA synthesis, end repair, a-base addition, and adaptor ligation following Illumina’s protocol. Subsequently, 15 PCR cycles using Phusion DNA polymerase (New England Biolabs, Ipswich, MA, USA) were employed for PCR amplification of cDNA target fragments measuring 200–300 bp. These paired-end libraries were sequenced on the NovaSeq 6000 platform (Illumina).

### Transcriptome sequencing and analysis

Raw reads were processed using Trimmomatic [22] (v.0.33), including trimming, the removal of low-quality reads, and adaptors. The resulting clean reads were aligned to the host reference genome (No. GCA_002263795.2) in orientation mode using the HISAT2 software [23] (v.2.2.0). To identify DEGs between the F and G groups, the expression levels of individual gene transcripts were estimated using the transcripts per million method. Subsequently, a differential expression analysis was conducted through the application of the R package edgeR [24] (v.3.32.1). Genes meeting the criteria of Benjamini–Hochberg adjusted FDR < 0.05 and log_2_FC (fold change) > 1 were recognized as true DEGs (differentially expressed genes). To elucidate the functions of these DEGs, we conducted Kyoto Encyclopedia of Genes and Genomes (KEGG) pathway analyses using KOBAS-i [25]. In detail, the targeted gene list was used as input, and the innovative landscape view in cirFunMap enabled downstream visualization of the output from the enrichment module (Correlation > 0.35 and top *n* = 7).

### Statistical analysis

Power analyses employing *t*-tests for the difference between two independent means calculated the minimum sample size with G*Power 3.1.9.7, guided by the effect size of PGE_2_ level in the hypothalamus. Our calculations showed that achieving a power of 0.8 at least 4 cows per group. Differences in days in milk, milk yield, body weight, and dry matter intake between the F and G groups were analyzed using the *t*-test, with significance determined at a threshold of *P* < 0.05. For the metabolome analysis, the comparison between the F and G groups was conducted using the Wilcoxon rank-sum test, with the differential abundance threshold set at *P* < 0.05 and log_2_FC > 1. The random forest model was further used to determine the key indicators of dietary changes using the "randomForest" package in R (v.4.1.2). The correlation of significantly changed metabolites in the hypothalamus, blood, and milk was based on Spearman’s correlation analysis.

## Results

### Changes in body weight and dry matter intake by the grain-based diet introduction

Compared with the forage-based diet, the grain-based diet showed no significant impact on body weight (*P* = 0.805) of dairy cows (Additional file 2: Fig. S1C). Moreover, dry matter intake was stable, with no significant differences observed during the first (*P* = 0.636), second (*P* = 0.658), and third weeks (*P* = 0.437) between the 2 groups (Additional file 2: Fig. S1D–F).

### Changes of hypothalamic metabolism by the grain-based diet introduction

To investigate the effects of grain intervention on hypothalamic metabolism in dairy cows, LC–MS was employed to analyze hypothalamic tissue. In total, 369 annotated metabolites were identified, including 82 fatty acyls, 78 organic acids, 21 nucleic acids, 20 sterol lipids, 19 organoheterocyclic compounds, 12 carbohydrates, 12 benzenoids, and 10 lipid-like molecules (Additional file 3: Fig. S2).

**Fig. 1:**
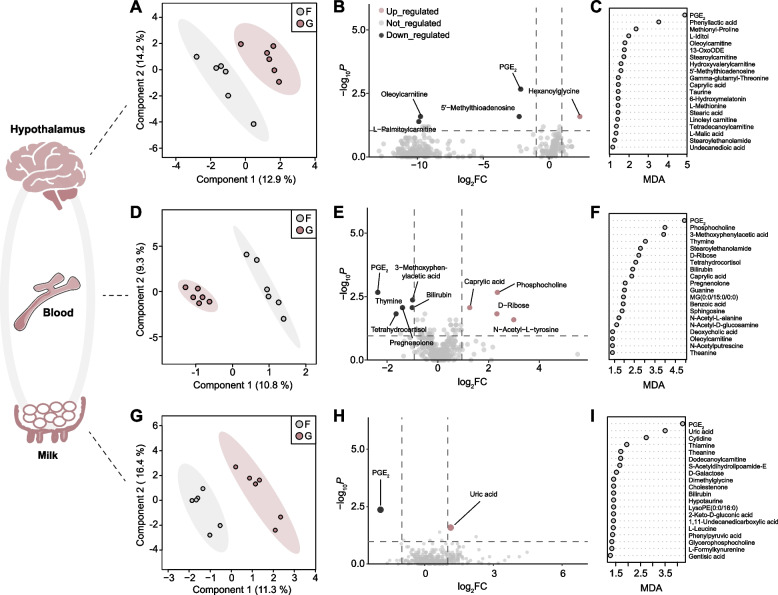
Altered metabolomic profiles in hypothalamic tissue, plasma, and milk of dairy cows following grain intervention. Sparse partial least-squares discriminant analysis (sPLS-DA) of metabolomic profiles in hypothalamic tissue (**A**), plasma (**D**), and milk (**G**) between the forage-based (F) and grain-based (G) diets (*n* = 6/group). Volcano plot of 369 metabolites in hypothalamic tissue (**B**), plasma (**E**), and milk (**H**). The red dots represent increased metabolites in the grain-fed cows (*P* < 0.05), the black dots represent decreased metabolites in the grain-fed cows (*P* < 0.05), and the gray dots represent no significantly changed metabolites in the grain-fed cows (*P* > 0.05) based on Wilcoxon rank-sum test. The top 20 metabolites with the strongest influence, presented in order of importance (top to bottom), based on the mean decrease accuracy (MDA) of the random forest analysis in hypothalamic tissue (**C**), plasma (**F**), and milk (**I**)

The sPLS-DA plot of the metabolite profile clearly demonstrated a significant differentiation between the forage-fed and grain-fed cows (Fig. 1A). For differential analysis of metabolites, five metabolites showed significant differences, including four that increased and one that decreased (Wilcoxon rank-sum test, *P* < 0.05 and log_2_FC > 1). In detail, the grain-based diet increased the level of hexanoylglycine (*P* = 0.026), while the levels of prostaglandin E_2_ (PGE_2_, *P* = 0.002), 5′-methylthioadenosine (*P* = 0.026), oleoylcarnitine (*P* = 0.026), and L-palmitoylcarnitine (*P* = 0.041) were decreased (Fig. 1B). Among them, PGE_2_ and L-palmitoylcarnitine were categorized as fatty acyls.

Furthermore, the random forest model was employed to identify the key indicator of grain intervention. Interestingly, PGE_2_ emerged as the distinctive hypothalamic metabolite in cows fed a grain-based diet, based on the mean decrease accuracy (Fig. 1C). Metabolic pathway analysis exhibited that PGE_2_ was an essential metabolite involved in arachidonic acid metabolism (Additional file 4: Table S2).

### Modifications in hypothalamic transcriptome profile following grain intervention

**Fig. 2:**
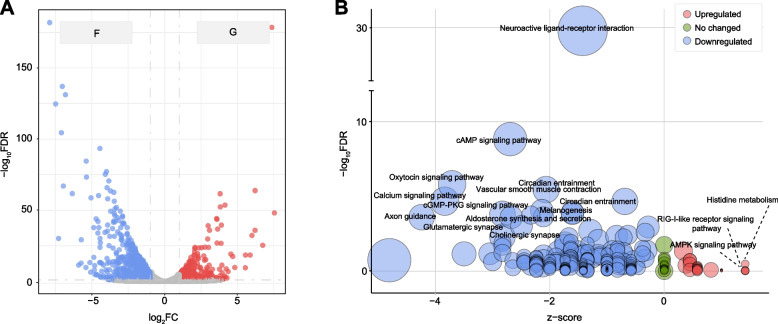
Effect of the grain-based diet on the hypothalamic transcriptome. **A** Volcano plots of differentially expressed genes (DEGs) between the forage-based (F) and grain-based (G) diets (edgeR, Benjamini–Hochberg adjusted FDR < 0.05 and log_2_FC > 1). Compared with the F group, the red dots represent significantly increased genes and the blue dots represent significantly decreased genes in the G group. **B** Kyoto Encyclopedia of Genes and Genomes (KEGG) pathway enrichment analysis of DEGs. Upregulated KEGG pathways are in red; unchanged KEGG pathways are in green; downregulated KEGG pathways are in blue. Z-score = (up − down)/count^½^, wherein, up, down, and count respectively represent the number of upregulated, downregulated, total DEGs in related KEGG pathways

To explore changes in the hypothalamic transcriptome profile linked to altered metabolism, we performed transcriptome sequencing on hypothalamic tissues. From the hypothalamic transcriptome, we generated a total of 494.9 million clean reads, with an average of 41.2 ± 1.8 million clean reads per hypothalamic sample. A total of 25,004 genes were detected, displaying an average expression exceeding 0.5 in at least one group. Within this pool of identified genes, 1,192 DEGs were noted in the comparison between forage and grain-fed cows (Fig. 2A). Specifically, the grain-based diet resulted in a significant upregulation of 363 genes and downregulation of 829 genes when compared to the forage-based diet (edgeR, Benjamini–Hochberg adjusted FDR < 0.05 and log_2_FC > 1).

KEGG pathway enrichment analysis of DEGs within hypothalamic transcriptome was further conducted. The grain-based diet led to a substantial increase in histidine metabolism, RIG-I-like receptor signaling pathway, and AMPK signaling pathway (Fig. 2B). Notably, the significant downregulation of numerous DEGs resulted in the suppression of various pathways, including neuroactive ligand-receptor interaction, calcium signaling pathway, oxytocin signaling pathway, cAMP signaling pathway, axon guidance, circadian entrainment, cGMP-PKG signaling pathway, vascular smooth muscle contraction, melanogenesis, glutamatergic synapse, aldosterone synthesis and secretion, and cholinergic synapse (Fig. 2B).

### Reconstruction of hypothalamic lipid metabolism by grain intervention

**Fig. 3:**
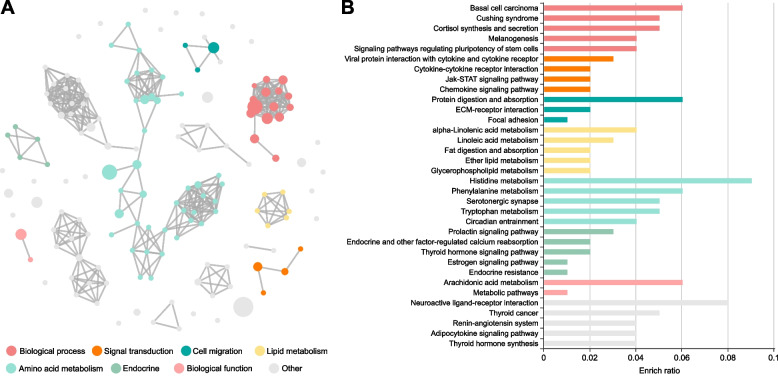
cirFunMap visualization of up-regulated KEGG pathways in the hypothalamus by grain intervention. **A** The node color represents different clusters, the node size represents *P*-value, and the edges represent correlations larger than 0.35. **B** Each row represents an enriched function, and the length of the bar represents the enrichment ratio, calculated as "input gene number"/"background gene number". The color of the bar corresponds to the color in the circular network above, representing different clusters

**Fig. 4:**
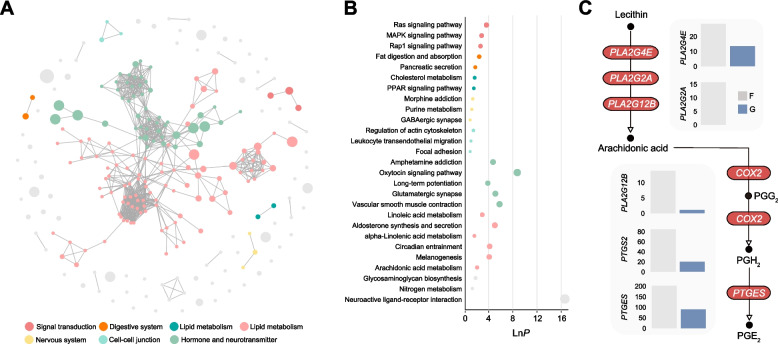
Functionally dysregulated lipid pathways in the hypothalamus by grain intervention. **A** cirFunMap visualization of the down-regulated KEGG pathways. The node color represents different clusters, the node size represents *P*-value, and the edges represent correlations larger than the 0.35. **B** Each bubble represents an enriched function. The color of the bar corresponds to the color in the circular network, representing different clusters. For each cluster, the top five functions with the highest enrichment ratio are displayed. **C** Arachidonic acid metabolism pathway in hypothalamic tissue. In the bar chart, gray bars represent the forage-based diet group (F), while the blue bars represent the grain-based diet group (G)

To explore whether the alterations in the hypothalamic metabolome are associated with the functionally dysregulated pathways, we performed gene set enrichment (GSE) analysis on the 363 upregulated DEGs and 829 downregulated DEGs, respectively. This analysis revealed 158 up-regulated and 244 down-regulated KEGG pathways, which were subsequently visualized using cirFunMap. The grain-based diet exhibited a significant increase in amino acid metabolism related to histidine metabolism, tryptophan metabolism, serotonergic synapse, and phenylalanine metabolism in hypothalamic tissues (Fig. 3A and B). Interestingly, down-regulated KEGG pathways in the grain-based diet group indicated a marked decrease in lipid metabolism (Fig. 4A). The primary terms within this cluster included linoleic acid metabolism, aldosterone synthesis and secretion, alpha-linolenic acid metabolism, circadian entrainment, and arachidonic acid metabolism, as highlighted by cirFunMap (Fig. 4A and B). These down-regulated pathways aligned with the observed reduction in the crucial indicator PGE_2_ after grain intervention.

### Grain-based diet decreases prostaglandin synthesis in the hypothalamus

Given this phenomenon, we hypothesized that the decrease in PGE_2_ in the hypothalamus is driven by functionally dysregulated pathways. Therefore, we further investigated the arachidonic acid metabolism and observed a significant reduction in the expression of phospholipase A2 (*PLA2G4E*, FDR < 0.001; *PLA2G2A*, FDR < 0.001; *PLA2G12B*, FDR = 0.002) in the grain-based diet group, leading to a decrease in the metabolic process from lecithin to arachidonic acid (Fig. 4C). Additionally, the expression of cyclooxygenase-2 (*COX2*; FDR < 0.001), which mediates the conversion of arachidonic acid to PGH_2_, was significantly decreased after the grain-based diet introduction. These alterations coincided with a noteworthy decrease in the expression of prostaglandin E synthase (*PTGES*; FDR < 0.001) in the grain-based diet group, resulting in a marked decrease in the level of PGE_2_. Therefore, our results suggest that the downregulation of arachidonic acid metabolism in the hypothalamus may be a crucial factor contributing to the decrease in the PGE_2_ level.

### Effect of grain intervention on blood metabolites

LC–MS analysis was also performed on plasma samples to examine alterations in peripheral circulation metabolites. A clear separation between forage and grain-fed cows was observed through sPLS-DA (Fig. 1D). Compared with the forage-based diet, the grain-based diet significantly increased the levels of phosphocholine (*P* = 0.002), N-acetyl-L-tyrosine (*P* = 0.026), D-Ribose (*P* = 0.015), and caprylic acid (*P* = 0.009) (Fig. 1E). In contrast, the levels of PGE_2_ (*P* = 0.002), tetrahydrocortisol (*P* = 0.015), pregnenolone (*P* = 0.009), thymine (*P* = 0.009), bilirubin (*P* = 0.009), and 3-methoxyphenylacetic acid (*P* = 0.004) showed the significant reduction in the grain-fed cows (Fig. 1E). Through random forest analysis, we identified PGE_2_, phosphocholine, and 3-methoxyphenylacetic acid as important metabolic indicators during dietary change, with PGE_2_ particularly displaying the highest mean decrease accuracy value (Fig. 1F).

### Effect of grain intervention on milk metabolites

LC–MS analysis was further performed on milk samples to examine metabolite changes. A distinct separation between forage and grain-fed cows was observed through sPLS-DA (Fig. 1G). Compared with the forage-based diet, the grain-based diet markedly elevated uric acid level (*P* = 0.026) and notably reduced PGE_2_ level (*P* = 0.004; Fig. 1H). Through random forest analysis, PGE_2_ was identified as the metabolite showing the most significant change (Fig. 1I).

### Correlation of significantly altered metabolites in the hypothalamus, blood, and milk

Spearman’s correlation analysis was performed to detect the potential relations in metabolic level among hypothalamus, blood, and milk. The results revealed strong correlations with a threshold of *P* < 0.05 (Additional file 5: Fig. S3). Hypothalamic PGE_2_ positively correlated with blood levels of tetrahydrocortisol, pregnenolone, PGE_2_, and 3-methoxyphenylacetic acid, but negatively with phosphocholine and D-Ribose (Additional file 5: Fig. S3A). Hypothalamic hexanoylglycine showed a positive correlation with D-Ribose in blood. Furthermore, hypothalamic 5′-methylthioadenosine was found to positively correlate with 3-methoxyphenylacetic acid and thymine in blood, while hypothalamic oleoylcarnitine and L-palmitoylcarnitine also positively correlated with thymine. Conversely, hypothalamic PGE_2_ and 5′-methylthioadenosine negatively correlated with uric acid in milk (Additional file 5: Fig. S3B). In milk, PGE_2_ positively correlated with blood levels of PGE_2_, 3-methoxyphenylacetic acid, thymine, and bilirubin, but negatively with phosphocholine and D-Ribose (Additional file 5: Fig. S3C). Milk uric acid showed a positive correlation with blood phosphocholine, N-Acetyl-L-tyrosine, and D-Ribose, but a negative correlation with thymine, bilirubin, PGE_2_, and 3-methoxyphenylacetic acid. These results indicate a significant correlation among metabolic changes in the hypothalamus, blood, and milk following a grain-based diet.

## Discussion

Hypothalamus plays an important role in regulating a broad spectrum of physiological processes, including the endocrine system, energy metabolism, dietary control, and microbial modulation [3, 4], highlighting the intricate link between hypothalamic function and the overall health and productivity of dairy cows. However, research concerning the hypothalamic functionality in dairy cows has been quite limited, especially in response to dietary interventions. Hence, our study employed untargeted LC–MS analysis and transcriptome sequencing to investigate the effects of feeding a grain-based diet on both hypothalamic metabolism and peripheral circulation in dairy cows.

In our LC–MS analysis, fatty acyls (22.22%) and organic acids (21.14%) emerged as primary components of the hypothalamic metabolome, together accounting for nearly half of the identified metabolites. Fatty acyls are integral components of lipid compounds, which are closely associated with many physiological processes, including energy storage, cell membrane composition, signal transduction, and metabolic regulation [26]. Lipid metabolism within the hypothalamus plays a vital role in maintaining energy balance and regulating various metabolic processes [27, 28]. Notably, our study found that a grain-based diet significantly altered the hypothalamic metabolome, especially impacting lipid compounds like PGE_2_. PGE_2_, a widely produced prostaglandin, is a biologically active lipid mediator produced from arachidonic acid through the action of cyclooxygenase and specific prostaglandin synthases [29–31]. Hypothalamic PGE_2_ is interconnected with its functions in peripheral tissues and is essential for regulating inflammatory response [32]. Previous studies have reported that PGE_2_ exists at nanomolar levels in most tissues at baseline, and its levels increase at sites of inflammation [33, 34]. Therefore, the reduced level of hypothalamic PGE_2_ observed in our study may suggest that the grain-based diet leads to a decrease in the inflammatory response. However, previous research has indicated that long-term feeding of high-grain diets promotes severe inflammatory responses in the gastrointestinal tract [10], liver [11], mammary gland [12], and other tissues of dairy cows. Considering our findings, one plausible interpretation is that the hypothalamus, in an effort to mitigate prolonged harm to tissues resulting from extended grain-based feeding, could tightly regulate the inflammatory response [35]. Consequently, following a severe inflammatory reaction, the hypothalamus might reduce PGE_2_ level as a means to alleviate the tissues’ inflammatory response. However, this speculation requires further experiments for validation.

Our analysis of the hypothalamic transcriptome revealed that a grain-based diet significantly increased amino acid metabolism, including pathways associated with histidine metabolism, tryptophan metabolism, serotonergic synapse, and phenylalanine metabolism in hypothalamic tissues. These metabolic pathways play a pivotal role in the synthesis and regulation of neurotransmitters, significantly influencing neurotransmitter function and associated physiological processes [36]. Thus, a grain-based diet may lead to increased synthesis and metabolism of hypothalamic neurotransmitters. In contrast, the grain-based diet resulted in a significant number of down-regulated genes, primarily associated with lipid metabolism pathways. These pathways encompassed linoleic acid metabolism, aldosterone synthesis and secretion, alpha-linolenic acid metabolism, circadian entrainment, and arachidonic acid metabolism. This observation is consistent with the hypothalamic metabolome results, highlighting a significant reduction in lipid metabolism resulting from the administration of grain-based diets. Further investigation of the lipid metabolism pathways revealed that a grain-based diet led to a significant downregulation in the expression of genes involved in the process of generating PGE_2_ from lecithin, including *PLA2G4E*, *PLA2G2A*, *PLA2G12B*, *COX2*, and *PTGES*. Phospholipase A2 is the primary enzyme responsible for generating arachidonic acid from phospholipids [37], and *COX2* is known to mediate the synthesis of prostaglandins, particularly under inflammatory conditions, where its levels rapidly increase [29, 32]. *PTGES* has been shown to be induced in a coordinated manner with *COX2*, indicating functional relevance [32]. Therefore, the downregulation of the hypothalamic arachidonic acid metabolism pathway may be a significant contributing factor to the reduction in PGE_2_ level.

It is intriguing to note that a grain-based diet exerts a profound influence on the peripheral circulation. Through the analysis of plasma and milk metabolomes, we observed a significant decrease in the level of PGE_2_ following grain-based diet feeding, which aligns with the results from the hypothalamic metabolome analysis. Moreover, hypothalamic PGE_2_ level were found to be positively correlated with blood levels of PGE_2_, tetrahydrocortisol, pregnenolone, and 3-methoxyphenylacetic acid. Tetrahydrocortisol is a downstream metabolite of cortisol, and pregnenolone is the precursor steroid in the biosynthesis of all steroid hormones [38]. Therefore, tetrahydrocortisol and pregnenolone are associated with hormone synthesis and metabolism in adrenal cortex, implying potential alterations in the HPA axis due to grain introduction. Previous research has suggested that hypothalamic PGE_2_ can regulate the HPA axis to modulate steroid hormone production [39, 40]. This implies that under a high-grain diet, hypothalamic PGE_2_ may regulate peripheral circulation metabolites through the HPA axis. Additionally, hypothalamic PGE_2_ and 5′-methylthioadenosine were found to negatively correlate with uric acid in milk. Uric acid, a product of purine metabolism, accounts for 21% of purine excretion by the mammary gland and acts as a significant radical scavenger, providing antioxidative protection against oxidative stress to cells [41]. Therefore, under a grain-based diet, fluctuations in hypothalamic PGE_2_ level are closely linked to mammary gland health. Consequently, the alterations in metabolites within the hypothalamus and peripheral circulation of dairy cows following grain-based diet feeding appear to be interconnected, yet further experiments are necessary to clarify this relationship.

## Conclusions

The introduction of a grain-based diet resulted in a significant reduction in hypothalamic PGE_2_ level, potentially due to the downregulation of the arachidonic acid metabolism pathway. Additionally, these was the substantial reduction in the level of PGE_2_ in the blood plasma, along with adrenal steroid hormones tetrahydrocortisol and pregnenolone. Analysis of the milk metabolome showed that the grain-based diet significantly increased uric acid level while notably decreasing PGE_2_ level. Correlation analysis highlighted a significant link between metabolic changes in the hypothalamus, blood, and milk after feeding a grain-based diet. Our findings indicate a potential relationship between changes in the hypothalamus and alterations in peripheral circulation following the introduction of the grain-based diet.

### Supplementary Information

**Additional file 1: Table S1.** Ingredients and nutritional compositions of the forage-based (F) and grain-based (G) diets.

**Additional file 2: Fig. S1.** Differences in DIM (**A**) and milk yield (**B**) between the two groups. Changes in body weight (**C**) and DMI (**D–F**) by the grain-based diet introduction. DIM, days in milk; DMI, dry matter intake.

**Additional file 3: Fig. S2.** Super chemical class sets of all hypothalamic metabolites in dairy cows.

**Additional file 4: Table S2.** Pathway enrichment analysis using significantly different metabolites between the forage (F) and grain-fed (G) cows.

**Additional file 5: Fig. S3.** Correlation of significantly changed metabolites in the hypothalamus, blood, and milk after feeding a grain-based diet. Only strong and significant correlations (*P* < 0.05) as determined by Spearman's correlation analysis are presented.

## Data Availability

Raw sequence reads for all samples are available under European Nucleotide Archive (ENA) project PRJNA723218 (SRR27392980-SRR27392991).

## Abbreviations

COX2: Cyclooxygenase-2
DEGs: Differentially expressed genes
ESI: Electrospray ionization
F: The forage-based diet
FC: Fold change
G: The grain-based diet
GSE: Gene set enrichment
HPA: Hypothalamic–pituitary–adrenal
KEGG: Kyoto Encyclopedia of Genes and Genomes
LC–MS: Liquid chromatography–mass spectrometry
PGE_2_: Prostaglandin E_2_
PLA2: Phospholipase A2
PTGES: Prostaglandin E synthase
sPLS-DA: Sparse partial least squares discriminant analysis
