# Supplementary material for: Effects of grain intervention on hypothalamic function and the metabolome of blood and milk in dairy cows
Source: J Anim Sci Biotechnol. 2024 Jun 1;15:71. doi: 10.1186/s40104-024-01034-3 (PMC11143652; doi:10.1186/s40104-024-01034-3)
Supplement: Supplementary file 1 — Additional file 1: Table S1. Ingredients and nutritional compositions of the forage-based (F) and grain-based (G) diets. [file 40104_2024_1034_MOESM1_ESM.docx]

**Additional file 1: Table S1** Ingredients and nutritional compositions of the forage-based (F) and grain-based (G) diets

| **Item** | **F** | **G** |
| --- | --- | --- |
| Ingredients, % of DM |  |  |
| Corn grain | 19.40 | 24.92 |
| Soybean | 13.50 | 13.48 |
| Barley | — | 12.00 |
| DDGS | 3.80 | 5.91 |
| CaCO_3_ | 0.80 | 1.48 |
| Ca(HCO₃)₂ | 1.10 | 0.92 |
| NaCl | 0.40 | 0.37 |
| Premix | 1.00 | 0.92 |
| Corn silage | 12.00 | 6.00 |
| American alfalfa hay | 24.00 | 17.00 |
| Australian oaten hay | 24.00 | 17.00 |
| Nutrients composition |  |  |
| DM, % | 46.77 | 48.03 |
| CP, % of DM | 16.16 | 16.12 |
| Crude fat, % of DM | 3.05 | 3.05 |
| NDF, % of DM | 36.14 | 29.92 |
| NFC, % of DM | 35.39 | 42.34 |
| Starch, % of DM | 17.96 | 27.82 |
| Ash, % of DM | 5.97 | 4.87 |
| Ca, % of DM | 1.14 | 1.18 |
| P, % of DM | 0.52 | 0.51 |
| NE_L_, Mcal/kg of DM | 1.57 | 1.64 |
| NFC/NDF | 0.97 | 1.42 |

Premix contained the following ingredients per kilogram of diet: vitamin A, 22.5 KIU/kg; vitamin D_3_, 5.0 KIU/kg; vitamin E, 37.5 IU/kg; vitamin K_3_, 5.0 mg/kg; Mn, 63.5 mg/kg; Zn, 111.9 mg/kg; Cu, 25.6 mg/kg; and Fe, 159.3 mg/kg
