# Supplementary material for: Effects of grain intervention on hypothalamic function and the metabolome of blood and milk in dairy cows
Source: J Anim Sci Biotechnol. 2024 Jun 1;15:71. doi: 10.1186/s40104-024-01034-3 (PMC11143652; doi:10.1186/s40104-024-01034-3)
Supplement: Supplementary file 2 — Additional file 2: Fig. S1. Differences in DIM (A) and milk yield (B) between the two groups. Changes in body weight (C) and DMI (D–F) by the grain-based diet introduction. DIM, days in milk; DMI, dry matter intake. [file 40104_2024_1034_MOESM2_ESM.docx]

**Additional file 2: Fig. S1** Differences in DIM (**A**) and milk yield (**B**) between the two groups. Changes in body weight (**C**) and DMI (**D**, **E**, and **F**) by the grain-based diet introduction. DIM, days in milk; DMI, dry matter intake

**
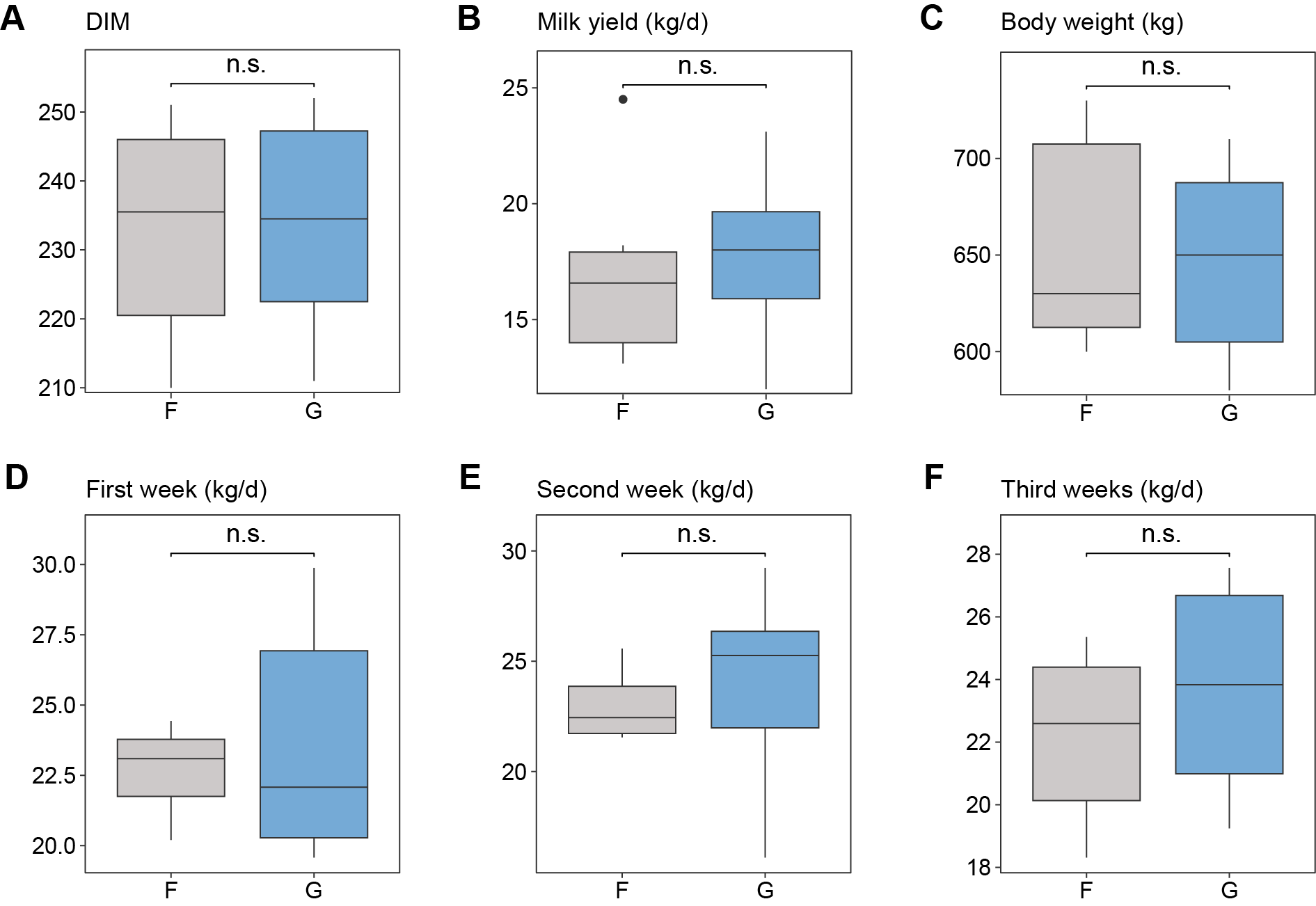
**
