# Supplementary figures and images for: Effects of grain intervention on hypothalamic function and the metabolome of blood and milk in dairy cows
Source: J Anim Sci Biotechnol. 2024 Jun 1;15:71. doi: 10.1186/s40104-024-01034-3 (PMC11143652; doi:10.1186/s40104-024-01034-3)

**Additional file 3: Fig. S2** Super chemical class sets of all hypothalamic metabolites in dairy cows.


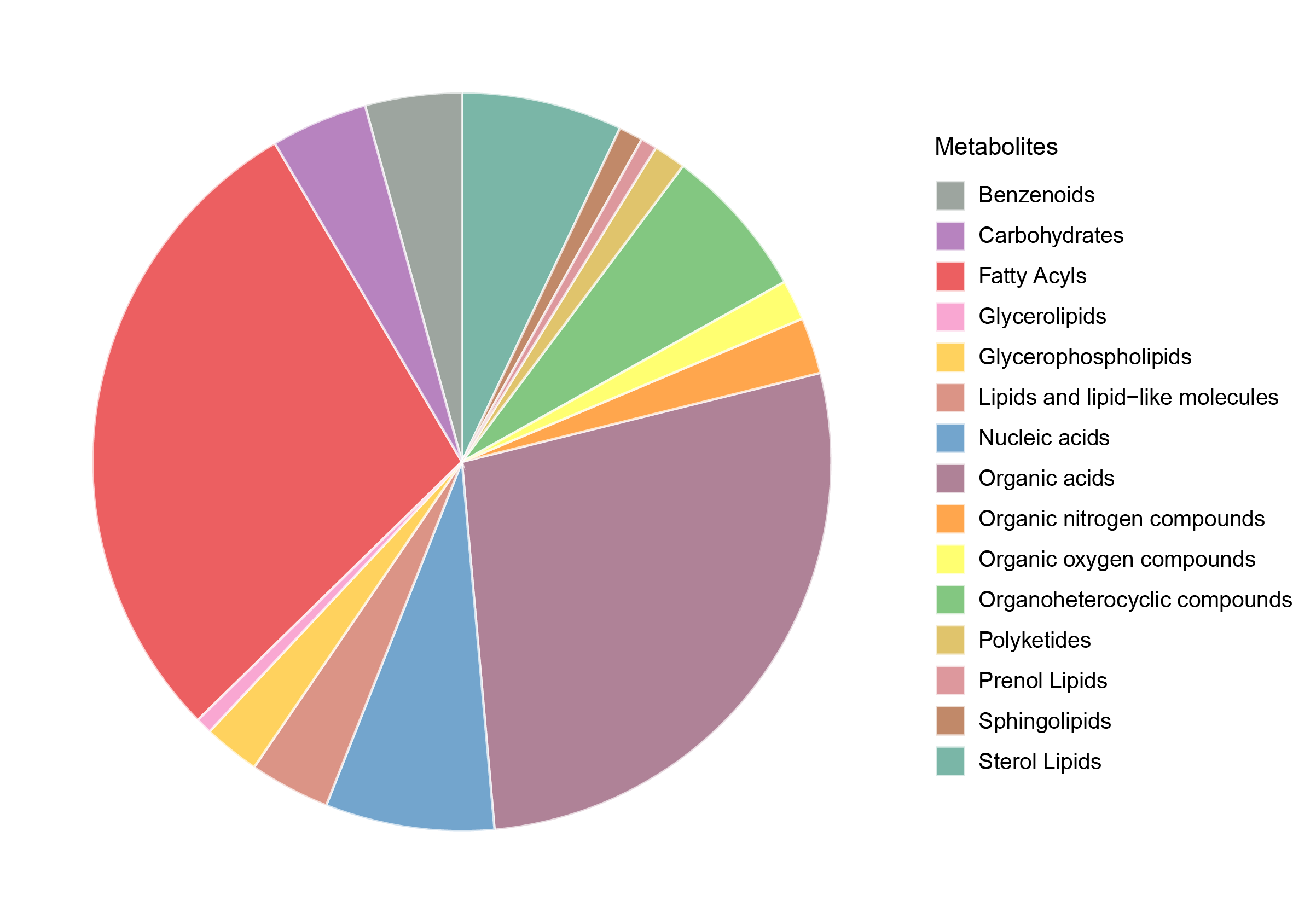

Supplement: Supplementary file 3 — Additional file 3: Fig. S2. Super chemical class sets of all hypothalamic metabolites in dairy cows. [file 40104_2024_1034_MOESM3_ESM.docx]
