# Supplementary material for: Effects of grain intervention on hypothalamic function and the metabolome of blood and milk in dairy cows
Source: J Anim Sci Biotechnol. 2024 Jun 1;15:71. doi: 10.1186/s40104-024-01034-3 (PMC11143652; doi:10.1186/s40104-024-01034-3)
Supplement: Supplementary file 4 — Additional file 4: Table S2. Pathway enrichment analysis using significantly different metabolites between the forage (F) and grain-fed (G) cows. [file 40104_2024_1034_MOESM4_ESM.docx]

**Additional file 4: Table S2** Pathway enrichment analysis using significantly different metabolites between the forage (F) and grain-fed (G) cows.

| **Pathway Name** | **Match** | **Metabolites** |
| --- | --- | --- |
| Arachidonic acid metabolism | 1/37 | PGE_2_ |
| Cysteine and methionine metabolism | 1/33 | 5'-Methylthioadenosine |
| Fatty acid degradation | 1/39 | L-Palmitoylcarnitine |
