# Supplementary material for: Effects of grain intervention on hypothalamic function and the metabolome of blood and milk in dairy cows
Source: J Anim Sci Biotechnol. 2024 Jun 1;15:71. doi: 10.1186/s40104-024-01034-3 (PMC11143652; doi:10.1186/s40104-024-01034-3)

**Additional file 5: Fig. S3** Correlation of significantly changed metabolites in the hypothalamus, blood, and milk after feeding a grain-based diet. Only strong and significant correlations (*P* < 0.05) as determined by Spearman's correlation analysis are presented.


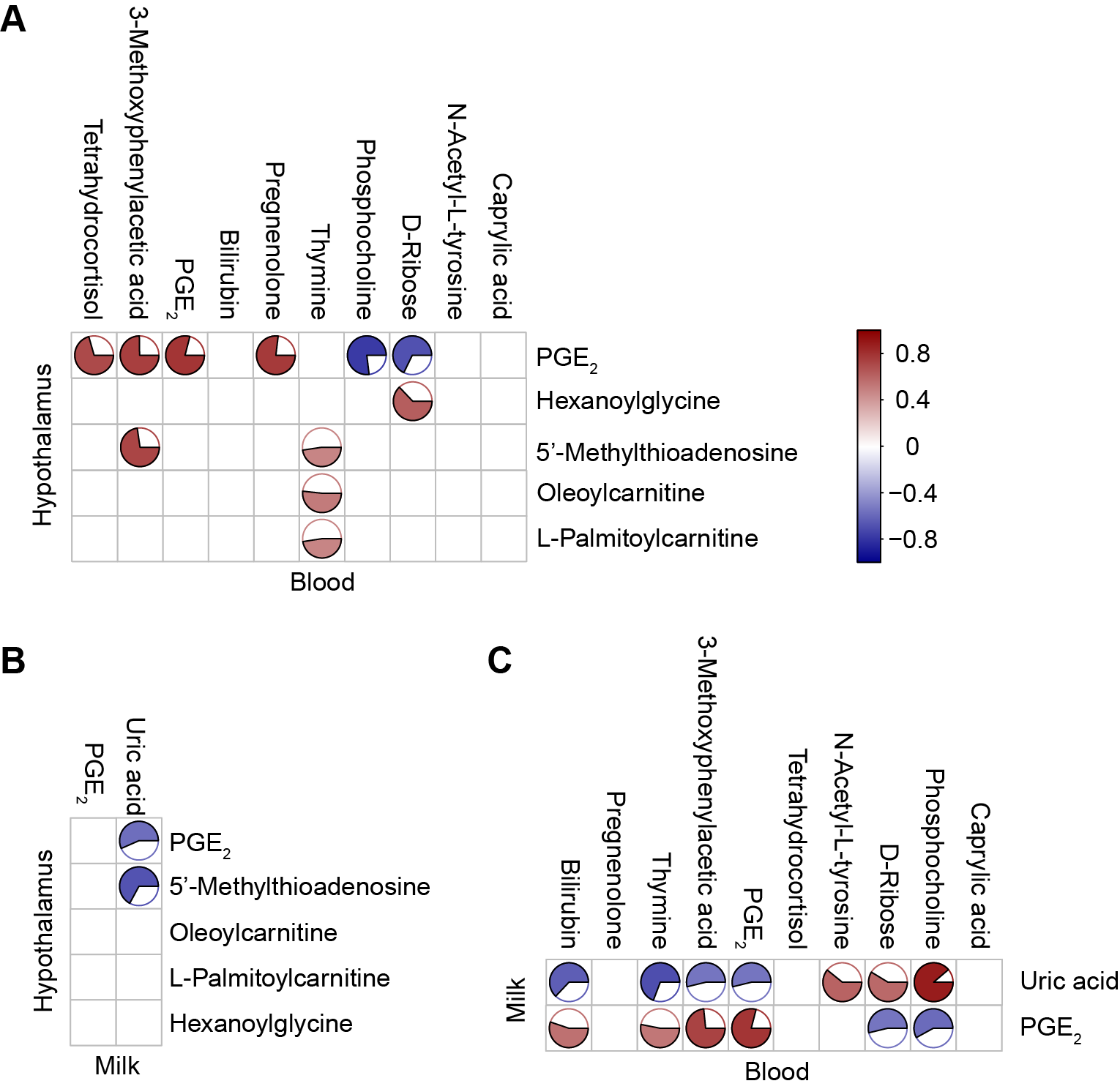

Supplement: Supplementary file 5 — Additional file 5: Fig. S3. Correlation of significantly changed metabolites in the hypothalamus, blood, and milk after feeding a grain-based diet. Only strong and significant correlations (P < 0.05) as determined by Spearman's correlation analysis are presented. [file 40104_2024_1034_MOESM5_ESM.docx]
